# Effect of Microwave-Assisted Heat–Moisture Treatment on Structure, Physicochemical Properties and In Vitro Digestibility of Wheat Starch

**DOI:** 10.3390/foods15101698

**Published:** 2026-05-12

**Authors:** Liuyan Chen, Jiawen Liu, Chao Yuan, Bo Cui

**Affiliations:** 1 Shandong Key Laboratory of Healthy Food Resources Exploration and Creation, Qilu University of Technology, Shandong Academy of Sciences, Jinan 250000, China; 19005415265@163.com; 2 School of Food Science and Engineering, Qilu University of Technology, Shandong Academy of Sciences, Jinan 250353, China; 3 Zaozhuang Dongliang Biotechnology Development Co., Ltd., Zaozhuang 277200, China; 13465973777@163.com

**Keywords:** microwave-assisted heat–moisture treatment, wheat starch, crystalline structure, chain length distribution, in vitro digestibility

## Abstract

Wheat starch serves as a major dietary carbohydrate. Optimizing its structural and functional properties is essential for developing health foods. In the present study, microwave-assisted heat–moisture treatment (MHT) was applied to modify wheat starch and the effects of the physical treatments on its structure and digestibility were investigated. X-ray diffraction analysis revealed that the crystallinity of wheat starch slightly decreased after MHT. Ion chromatography revealed changes in the chain length distribution of wheat starch after modification, with a continuous increase in short-chain components over treatment time. MHT enhanced the enzymatic resistance of wheat starch, which resulted in a resistant starch content of 36.89% after 1.5 h of MHT. Excess heat disrupted the ordered structure of starch when the treatment was extended to 2 h, leading to a slight reduction in enzymatic resistance. The study provided a theoretical basis for designing functional starch ingredients through low water content physical treatment.

## 1. Introduction

Starch serves as a primary energy source in the human diet, and its digestive properties have a significant impact on health [1]. Conventional starch undergoes rapid enzymatic hydrolysis in the digestive tract, where it is converted into glucose and quickly absorbed. This process not only triggers pronounced postprandial blood glucose spikes but may also increase the risks of obesity, diabetes, and cardiovascular disease [2]. Resistant starch cannot be digested or absorbed in the small intestine. Instead, it passes into the large intestine, where microorganisms ferment it, producing short-chain fatty acids [3]. The mechanism helps slow the rise in blood glucose after meals, potentially offering therapeutic benefits for individuals with diabetes and other metabolic conditions [4]. Therefore, consuming starchy foods rich in resistant starch serves as an effective strategy to mitigate starch digestion-related diseases and improve gut microbiota composition. RS is typically classified into five types based on its characteristics and origins: RS1 (physically embedded starch), RS2 (native granular), RS3 (retrograded starch), RS4 (chemically modified starch), and RS5 (amylose–lipid complex) [5,6]. Among these, RS3 is the primary type produced by physical modification methods. RS3 can form either by retrogradation of gelatinized starch upon cooling or through direct physical modification. In both processes, the starch chains rearrange into double-helical structures, thereby resisting enzymatic hydrolysis [7,8].

As one of the primary starch sources in daily diets, wheat starch contributes over 20% of global caloric intake [9]. Optimizing its functional properties is crucial for improving public health. However, compared to other common starches, such as corn starch, in-depth research on wheat starch, particularly the regulatory mechanisms of its digestive properties, remains relatively limited.

Contemporary wheat starch modification techniques include physical, chemical, and enzymatic methods [10]. Physical modification has garnered significant attention due to its simplicity, lack of chemical reagents (aligning with the “clean label” trend), and its ability to effectively alter starch structure and function [11]. Common physical modification techniques include heat–moisture treatment, microwave treatment, ohmic heating, and dry heat treatment [12,13]. Microwave-assisted heat–moisture treatment (MHT) is a promising technology. Its rapid and uniform heating mechanism allows for precise reorganization of starch granules, promoting the generation of resistant starch [14]. Recent studies have confirmed that this physical modification technique significantly reduces starch digestibility. For example, Frasson et al. [15] demonstrated that microwave-assisted heat–moisture treatment with 8% avocado oil significantly increased resistant starch content in rice starch and lowered the estimated glycemic index to 72.99 (*p* < 0.05). Jiang et al. [16] optimized a combined autoclave–microwave technology with a temperature-cyclic regeneration process and promoted double-helix rearrangement, which led to a 39.27% resistant starch content in high-amylose corn starch. These results collectively validate MHT as a scalable strategy for developing low-glycemic-index starch materials. Most current studies have focused on modifying starch using MHT at high water content, where starch is presented as a paste [17,18]. However, few researchers have explored the impact of MHT on wheat starch at low water content. We hypothesized that MHT could effectively increase the resistant starch content of wheat starch by promoting double-helix rearrangement under low water content.

The aim of the study is to modify low water content wheat starch through MHT, primarily to increase its resistant starch content. Additionally, changes in the properties of the starch will be investigated, including X-ray diffraction pattern, FTIR spectrum, chain length distribution, thermal properties, pasting characteristics, rheological behavior, in vitro digestibility and morphological properties. The study will establish a theoretical foundation for the microwave-assisted heat–moisture treatment of wheat starch, and could advance the development of healthy products with a high resistant starch content.

## 2. Materials and Methods

### 2.1. Materials

Wheat starch (botanical source: wheat (Lumai 23); supplier catalog number: ZZ25SW1720505A; amylose content: 23.1%; protein: 0.32%; lipids: 0.03%; ash: 0.23%; moisture: 12.2%; Mw: 2.83 × 10^6^ g/mol) was obtained from Zaozhuang Dongliang Biotechnology Co., Ltd. (Zaozhuang, China). The weight-average molecular weight (Mw) of the wheat starch was determined using a high-performance size exclusion chromatography system coupled with differential refractive index and multi-angle laser scattering (HPSEC-MALS-RI, Wyatt Technology Co., Santa Barbara, CA, USA) [19]. α-amylase (from porcine pancreas, 100 U/mg) was purchased from Sigma-Aldrich (Shanghai, China). Amyloglucosidase (106 U/mL) was purchased from Shanghai Yuanye Bio-Technology Co., Ltd. (Shanghai, China). All other chemical reagents were of analytical grade.

### 2.2. Microwave Treatment (MT)

The wheat starches were mixed with deionized water to adjust the water content of the starch samples to 20%. The starch sample, evenly spread in a glass container (approximately 14 cm in length and 9 cm in width), was treated in a microwave oven (M3-L236E, Guangdong Midea Kitchen Appliance Manufacturing Co., Ltd., Foshan, China) at 2.45 GHz and 900 W for 3 min. After being refrigerated at 4 °C for 24 h, the sample was dried at 40 °C for 24 h. It was then ground and sieved through a 100-mesh screen. The obtained starch sample was marked as MTS.

### 2.3. Microwave-Assisted Heat–Moisture Treatment (MHT)

The wheat starches were first subjected to microwave irradiation following the same procedure described in Section 2.2. Following microwave treatment, the water content of the starch was readjusted to 20%. The samples were individually placed in a hydrothermal autoclave reactor and treated in an oven at 100 °C for 0.5 h, 1 h, 1.5 h and 2 h, respectively. After being refrigerated at 4 °C for 24 h, the samples were dried at 40 °C for 24 h. Finally, they were ground and sieved through a 100-mesh screen. The resulting starch samples were designated as MHT-0.5 h, MHT-1 h, MHT-1.5 h and MHT-2 h, respectively.

### 2.4. Scanning Electron Microscopy (SEM)

Starch samples were uniformly sprinkled onto double-sided tape, and excess starch was gently blown off using an ear syringe. The samples were then sputter-coated with gold. Granule morphology was observed using a scanning electron microscope (SEM, Regulus 8220, Hitachi, Tokyo, Japan).

### 2.5. X-Ray Diffraction (XRD)

A wide-angle X-ray diffractometer (XRD-3D, Beijing, China) was used to analyze the crystallinity of the samples. The scanning was performed over a diffraction angle (2θ) range of 4° to 40° at a rate of 20°/min. Relative crystallinity was calculated using MDI Jade 6 software.

### 2.6. Fourier Transform Infrared Spectroscopy (FTIR)

Changes in the short-range ordered structure of the starches were analyzed using an FTIR Spectrometer (PerkinElmer, Shelton, CT, USA). The scan wavenumber range was 4000 cm^−1^ to 400 cm^−1^, with 32 scans and a resolution of 4 cm^−1^. The infrared spectra absorbance data from 800 to 1200 cm^−1^ were deconvoluted by OMNIC 9.2 software (enhancement factor: 1.9; peak width: 38 cm^−1^). R_1050/1020_ and R_1020/990_ were obtained from the peak intensity ratio of 1050 cm^−1^ and 1020 cm^−1^, and 1020 cm^−1^ and 990 cm^−1^, respectively.

### 2.7. Chain Length Distributions of Amylopectin

The chain length distribution of the starches was determined using ion chromatography (DIONEX ICS-5000+, Thermo Fisher Inc., Waltham, MA, USA), following a previously reported method with slight modifications [20]. A 0.5% (*w*/*v*) starch dispersion was prepared in sodium acetate buffer (0.1 M, pH 4.5). The mixture was boiled for 25 min and then cooled to 40 °C. Debranching was initiated by adding 10 μL of isoamylase. After the reaction, the sample was diluted with 0.15 M NaOH to 0.2% (*w*/*v*). The solution was then injected via a filter membrane into the HPAEC system. The instrument was operated at a flow rate of 0.5 mL/min. Chromatograms were processed using Chromeleon 7 software (Thermo Fisher Scientific, Waltham, MA, USA).

### 2.8. Differential Scanning Calorimetry Measurement (DSC)

The thermal properties of starch samples were analyzed using a differential scanning calorimeter (Netzsch Scientific Instruments, Selb, Germany). A total of 3 mg of starch was weighed into an aluminum sample pan, and deionized water was added to achieve a water-to-starch volume ratio of 3:1 (*v*/*w*). The sealed pans were equilibrated and then heated from 20 °C to 90 °C at a rate of 10 °C/min. The onset temperature (T_0_), peak temperature (T_p_), end temperature (T_c_), temperature range (ΔT) and enthalpy change (ΔH) data were processed with the NETZSCH Proteus Thermal Analysis Software 8.0.

### 2.9. In Vitro Digestibility

The starch samples (20 mg) were suspended in 17.5 mL of acetic acid–sodium acetate solution (0.2 M, pH = 5.2), and then the mixture was placed in a 90 °C water bath for 15 min. After cooling to 37 °C, 5 mL of mixed enzyme solution (containing 290 U/mL of α-amylase and 30 U/mL of amyloglucosidase) was added to each tube. The mixture was hydrolyzed at 37 °C with a stirring rate of 150 r/min, and 25 μL of the mixed solution was collected at 0 min, 20 min, 40 min, 60 min, 120 min, 180 min, 240 min, and 300 min of enzyme hydrolysis, respectively. The glucose content produced from the hydrolysis of the samples was determined using an SBA-40D biosensing analyzer (Shandong Academy of Sciences, Jinan, China). The fractions of rapidly digestible starch (RDS), slowly digestible starch (SDS) and resistant starch (RS) were calculated according to the following formulas:

RDS (%) = 0.9 × (G20 − G0)/TS × 100%,(1)

SDS (%) = 0.9 × (G120 − G20)/TS × 100%,(2)

RS (%) = 100 − RDS% − SDS%(3)

G20 and G120 represent glucose values released at 20 min and 120 min, respectively. G0 denotes free glucose content and TS indicates the total starch content of the sample.

The digestion kinetics of starch were modeled using the following model.

C_t_ = C_∞_ × (1 – e^−*k*t^) (4)

where C_t_ represents the degree of starch hydrolysis at time t, C_∞_ denotes the predicted percentage of starch digestion at the reaction endpoint, and *k* is the rate constant for in vitro digestion.

### 2.10. Pasting Properties

The gelatinization characteristics of the samples were analyzed with a Rapid Viscoanalyst (RVA, Newport Scientific, Warriewood, Australia). Starch (2.5 g) was dispersed in deionized water to reach a total suspension weight of 28 g. The sample was first equilibrated at 50 °C for 1 min, heated to 95 °C at 12 °C/min, and held at 95 °C for 2.5 min. It was then cooled back down to 50 °C at 12 °C/min and held at 50 °C for 2 min.

### 2.11. Rheological Properties

The apparent viscosity of the starch was measured using a rheometer (Physica MCR 302, Anton Paar GmbH, Graz, Austria) following the method described by Sun et al. [21] with minor modifications. A 10% (*w*/*v*) starch suspension was heated in a 95 °C water bath for 15 min and then equilibrated at 25 °C for 20 min. Steady-state shear tests were conducted at 25 °C over a shear rate range of 1–100 s^−1^.

### 2.12. Statistical Analysis

Data were analyzed using one-way analysis of variance (ANOVA) with SPSS 27.0 software. Values of *p* < 0.05 were considered statistically significant. All measurements were performed in triplicate and the Waller–Duncan post hoc test was used for multiple comparisons.

## 3. Results

### 3.1. SEM

**Figure 1:**
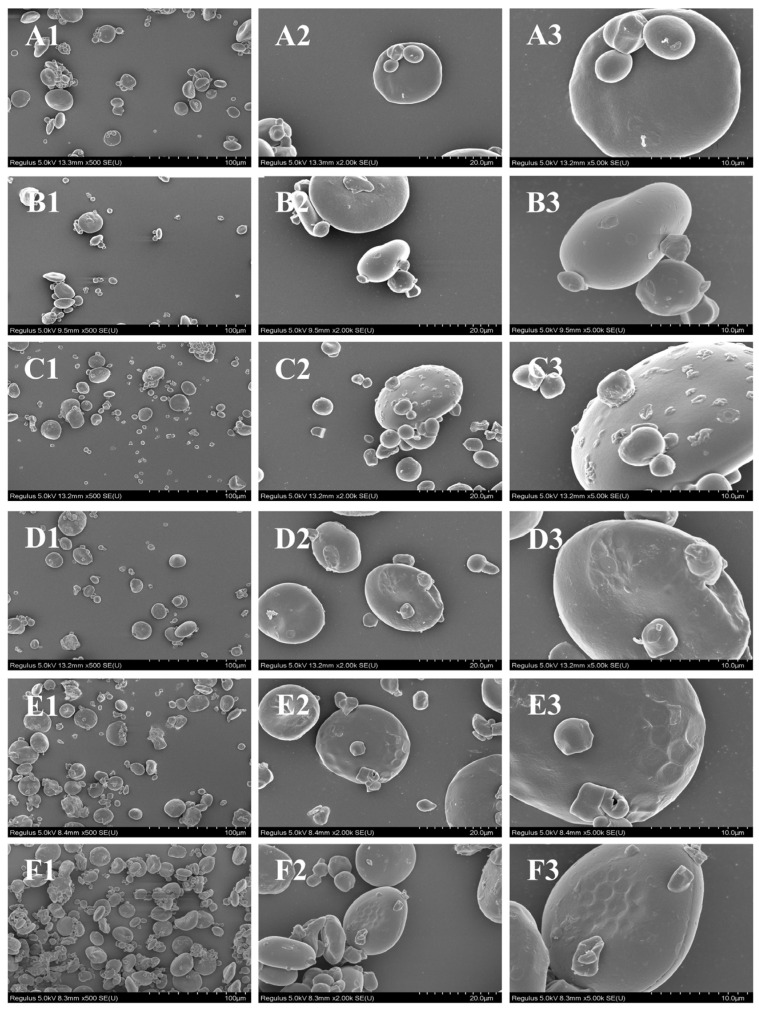
SEM images of WS and treated starch at 500× (**1**), 2000× (**2**) and 5000× (**3**) magnification. WS (**A**); MTS (**B**); MHT-0.5 h (**C**); MHT-1 h (**D**); MHT-1.5 h (**E**); MHT-2 h (**F**).

Morphological changes in the wheat starch were observed using SEM. As demonstrated in Figure 1, wheat starch granules comprise two types, type A and type B. Type A granules are large, with diameters ranging from 10 to 40 μm, while type B granules are small, with diameters less than 10 μm [22]. Native wheat starch exhibited a smooth surface and an intact granular structure [23]. In contrast, microwave-treated starch granules exhibited relatively rougher surfaces, likely attributable to the thermal effects and penetration of microwave energy [24]. The morphology and surface of MHT-modified starch was affected to varying degrees by different MHT durations. The starch granule surface became rougher than that of MTS and some particles adhered to each other after 0.5 h of MHT.

For MHT-1 h and MHT-1.5 h, the granules exhibited surface depressions and significant aggregation. The results were consistent with previous findings [25]. During the MHT process, the high temperature induced starch granules to absorb more water, leading to swelling and the overflow of some irregular starch fragments [26,27,28,29]. Upon completion of the MHT process, the subsequent cooling caused the granules to collapse, resulting in the observed surface depressions. The aggregation of starch granules into clusters was due to the expansion of branched starch and the escape of amylose during MHT [30]. When the MHT duration reached 2 h, the MHT-modified starch granules began to melt, and their surfaces became rougher. This was correlated with the prolonged MHT of the starch, which caused the starch granules to swell and disintegrate [31].

### 3.2. X-Ray Diffraction

**Figure 2:**
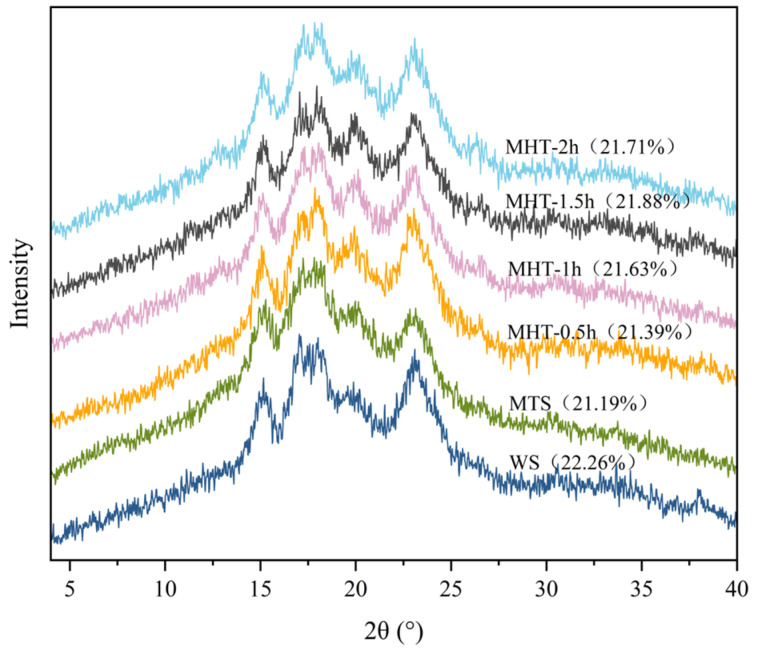
The X-ray diffraction pattern of native starch and treated starch samples.

As shown in Figure 2, the distinct diffraction peaks observed at 15°, 17°, 18° and 23° characterize the typical A-type X-ray diffraction pattern of wheat starch [32]. The intensity of these characteristic peaks decreased after MT. Similar results were reported by Deka and Sit [33], who observed that microwave-modified starch exhibited a significant reduction in peak intensity. The thermal effects of MT disrupted the microcrystalline arrangement of starch, and the heat generated by dipolar rotation of water molecules under microwave irradiation damaged starch chains, which led to molecular disorder and reduced the relative crystallinity of starch granules [34].

The crystallinity of all MHT-modified samples was slightly lower than that of native starch, which aligns with previous reports [33,35,36,37]. However, the differences in crystallinity among samples treated for different durations were minor. Overall, MHT had a limited impact on the long-range crystalline order of the starch, and the influence of treatment time was not significant.

### 3.3. Fourier Transform Infrared Spectroscopy

**Figure 3:**
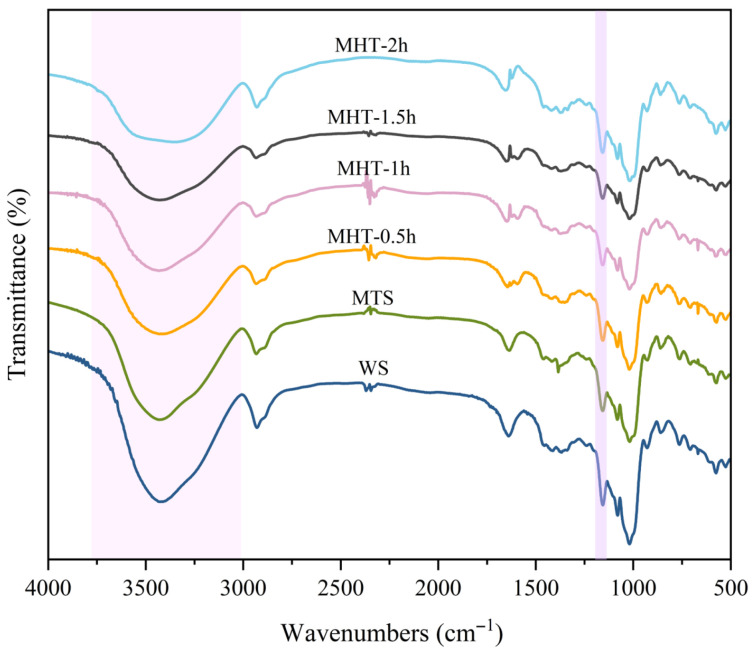
The FTIR spectra of native starch and modified starch samples.

The absorption band in the 3000–3750 cm^−1^ range corresponds to the O-H stretching vibration, the intensity of which reflects the strength of hydrogen bonding (Figure 3). The intensity of this peak weakened in the wheat starch following both MT and MHT. The peaks further diminished and narrowed as MHT duration increased, suggesting that both the MT and MHT processes disrupted the double-helix structures and hydrogen bonds. Additionally, the decreased intensity of the C-C stretching vibration at 1163 cm^−1^ indicated the degradation of starch chains following modification [26].

The absorption bands at 1050, 1020, and 990 cm^−1^ correspond to the crystalline region, amorphous region and short-range order structure of starch, respectively [38,39,40]. The ratios R_1050/1020_ and R_990/1020_ can be used to evaluate the short-range molecular order and the helix content in starch, respectively [41,42,43]. Both MTS and MHT-modified starch exhibited higher R_1050/1020_ and R_990/1020_ values than native starch (Table 1). The microwave thermal effect and heat–moisture treatment both promoted rearrangement of starch crystal regions under low water content conditions, which improved the double-helix structure and enhanced the short-range order of treated starch [34,44].

Within the period of time from 0.5 h to 1.5 h, both the R_1050/1020_ and R_990/1020_ values of the MHT-modified starch increased monotonically and reached their maximum values at 1.5 h. With the further extension of MHT time to 2 h, both ratios decreased significantly. This indicated that excessive treatment disrupted the double-helix structure and reduced the short-range molecular order [31]. Notably, these results differ slightly from the XRD data. This discrepancy may be explained by the fact that FT-IR reflects the short-range order associated with double-helix content, whereas XRD reveals the long-range order related to the packing of double helices [25,45].

### 3.4. Chain Length Distribution Analysis

**Figure 4:**
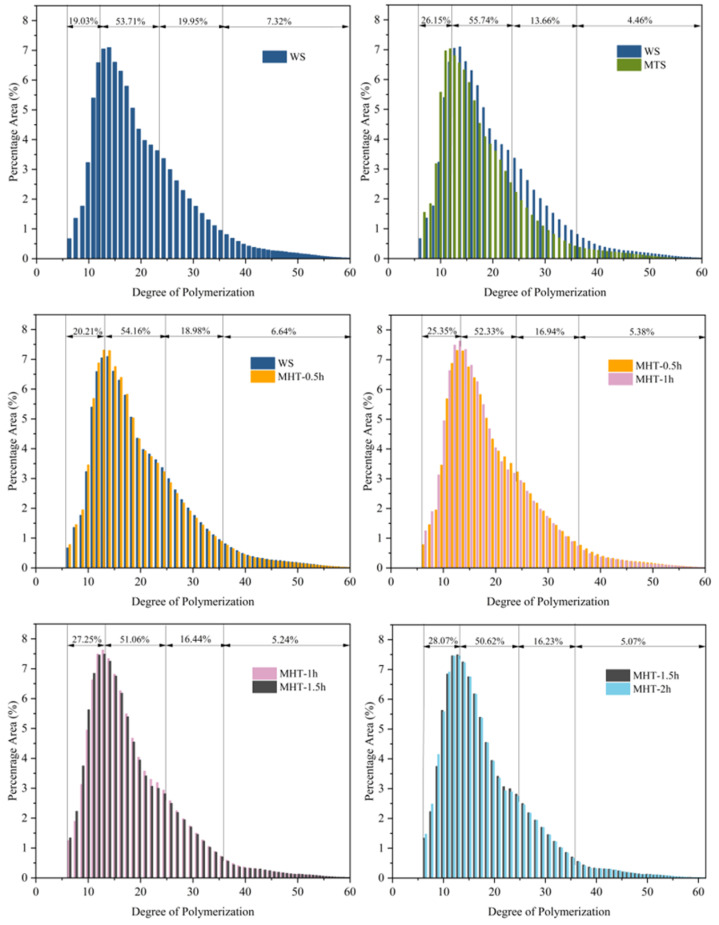
The chain length distribution of native starch and treated starch samples.

Based on the amylopectin clustering model, starch chains were classified into four categories: A chains (6 ≤ DP < 12), B1 chains (12 ≤ DP < 24), B2 chains (24 ≤ DP < 36), and B3 chains (36 ≤ DP < 100) [46]. The results indicated that compared to native starch, MTS exhibited increased A chains (short chains) and decreased percentages of B1, B2, and B3 chains (Figure 4). This suggested a shift toward shorter chains after microwave treatment. The proportion of A chain in MHT-modified starch progressively increased with extended MHT modification time and reached a peak of 35.52% after 2 h of modification. This indicated subsequent heat–moisture treatment also broke the starch chains. Zheng et al. [47] have reported similar results that the ratio of short chains increased in wheat starch after annealing and thermal-moisture treatment, while the ratios of medium length chains and long chains both decreased. The persistent thermal effects during MHT modification caused the breakage of starch chains and consequently increased the content of short chains. These newly formed short chains migrated and rearranged during MHT. In addition, FTIR analysis revealed an increase in short-range molecular order [2,48,49].

### 3.5. Thermal Properties

The DSC thermograms of all samples displayed a single endothermic peak within the temperature range of 57 °C to 74 °C, which corresponded to the gelatinization process (the DSC thermograms are presented in Figure S1). For native starch, the values of T_0_, T_p_, T_c_ and ΔH were 57.5 °C, 62.6 °C, 66.9 °C and 9.5 J/g, respectively (Table 2). MTS exhibited slightly lower gelatinization peak temperatures and enthalpy values compared to native starch, at 61.93 °C and 6.57 J/g, respectively. Liu, Wen, Zhang, Wang, Song and Chang [34] also reported similar results. Microwave treatment disrupted the long-range crystalline order of native starch and facilitated the rearrangement of crystalline domains, which led to the formation of double-helix structures with reduced stacking perfection. It contributed to the lower gelatinization temperature and enthalpy.

MHT-modified starch exhibited higher gelatinization peak temperatures than native starch. The T_p_ of MHT-modified starch increased progressively with extended MHT duration and reached 70.97 °C after 2 h. The increase in T_p_ suggested that the remaining or newly formed ordered structures after MHT exhibited higher thermal stability compared to native starch. Meanwhile, the enthalpy value of MHT-modified starch decreased progressively after MHT. The reduction reflected a loss of long-range ordered structures, consistent with the slight decrease in total crystallinity observed via XRD. Furthermore, the observed increase in T_p_ and decrease in ΔH were consistent with previous studies on heat–moisture-treated starch [50,51]. MHT selectively disrupted less stable ordered regions while preserving or forming thermally more stable ordered structures (e.g., double helices), as further supported by the FTIR and chain length distribution data [52,53,54].

### 3.6. In Vitro Digestibility

**Figure 5:**
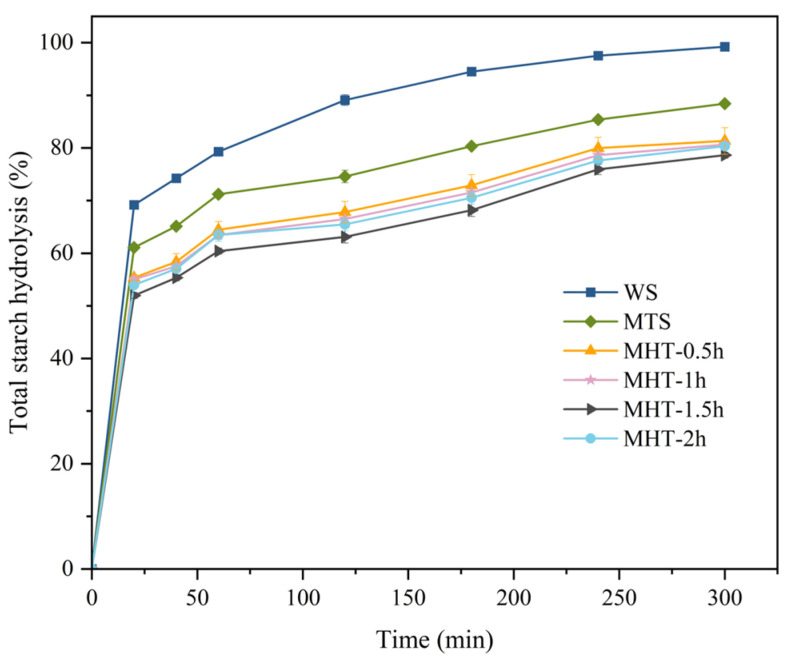
Hydrolysis curves of native starch and modified starch samples.

The hydrolysis curves and digestion characteristics of starch are shown in Figure 5 and Table 3, respectively. All samples exhibited rapid digestion rates during the initial phase (0 min–20 min), which subsequently slowed due to substrate depletion. Native starch reached a hydrolysis degree of 99.22% at 300 min, with RDS, SDS, and RS contents of 69.19%, 18.89%, and 11.92%, respectively. The hydrolysis degree of MTS decreased to 88.42% at 300 min, accompanied by an increase in RS content. Both the C_∞_ value and *k* value of MTS were lower than those of native starch, which indicated that both the degree of hydrolysis and hydrolysis rate of MTS were inhibited.

The RDS and SDS contents of starch decreased after MHT, while the RS content increased significantly, which was consistent with previous research results [29,55,56]. The RS content of the MHT-modified starch initially increased and then decreased with extended MHT duration, reaching a peak of 36.89% at 1.5 h. Concurrently, the C_∞_ value and *k* value also dropped to their lowest levels at this time, recorded as 69.78% and 0.0524, respectively. The hydrolysis extent and hydrolysis rate of MHT-modified starch were lower than those of both native starch and MTS.

As reported by Li et al. [57], the rearrangement of molecular chains and the formation of ordered structures, such as locally ordered regions and changes in thermodynamic properties, contributed to enhanced resistance against enzymatic action. MHT caused degradation of starch chains and partially disrupted the native helical and crystalline structures of starch. After these structures were disassembled, starch chains reassembled, forming new helices and ordered structures. These structures hindered the penetration of enzymes into the starch matrix, thereby increasing the resistance of starch to enzymatic digestion [58].

When MHT was extended to 2 h, the RS content significantly decreased to 34.52%, while the C_∞_ and *k* values increased to 71.81% and 0.0541, respectively. Sun et al. [59] also reported similar results. The change was attributed to the degradation or depolymerization of some ordered structures caused by excessive thermal treatment, which reduced the RS content and increased the digestibility of starch.

### 3.7. Pasting Properties

The gelatinization characteristics of modified starches exhibited significant differences compared to native starches (*p* < 0.05), as shown in Table 4 (the RVA pasting curves are presented in Figure S2). MTS showed a significantly lower peak viscosity than native starch. The integrity of starch granules was disrupted during microwave modification, which reduced their water absorption and swelling capacities [60]. All MHT-modified starches exhibited lower peak viscosity (PV), trough viscosity (TV), break down (BD), final viscosity (FV), and setback (SB) values than both native starch and MTS. Additionally, their pasting temperature (PT) values were higher than native starch. A tight structure formed within the starch granules following MHT. The durability of starch throughout the heating process was enhanced by the tight structure and the swelling rate was reduced [31].

MHT reduced the expansion capacity of starch more significantly than microwave modification, as it induced starch granules to become structurally tighter and inhibited swelling. Furthermore, the PV and TV values of MHT-modified starch decreased with increasing MHT duration, which was due to starch granule reorganization and reduced water absorption following MHT [61]. Consistent with the peak viscosity trends, the breakdown, final viscosity, and setback values of MHT-modified starch also decreased as MHT duration increased. The result could be attributed to the rearrangement of amylose molecules leached from swollen granules [62].

### 3.8. Rheological Properties

**Figure 6:**
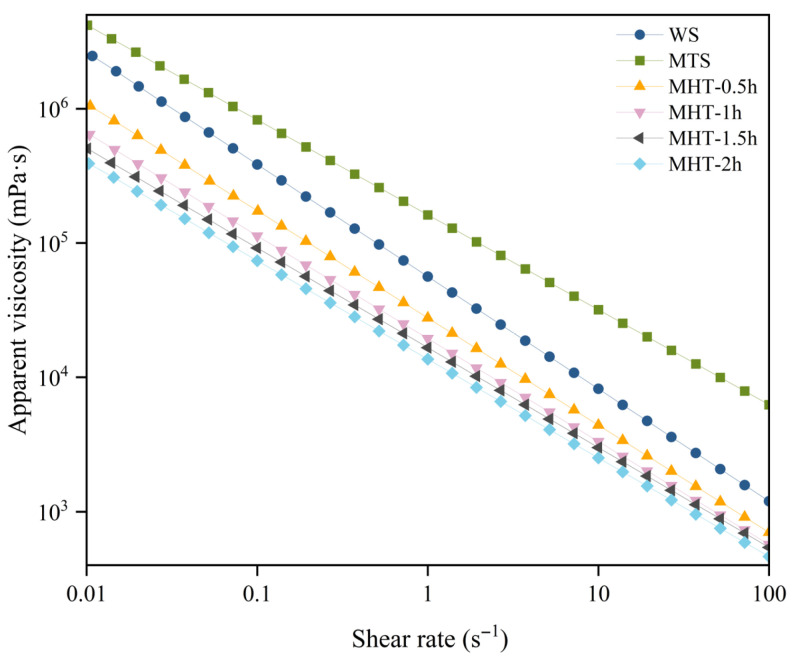
The rheological properties of native starch and modified starch samples.

Figure 6 shows the viscosity–shear rate curves for the samples. All wheat paste samples exhibited shear thinning behavior, as their apparent viscosity decreased with increasing shear rate, consistent with previous research results [28,29]. Under shear action, the conformation of polymer chains changed from their normal state, which gradually untangled the entangled polymer segments. This reduced the forces between segments and subsequently lowered viscosity [63]. The apparent viscosity of MTS was higher than that of native starch. Microwave treatment caused partial breakage of starch chains. During cooling after gelatinization, these shorter chain segments reassociated more readily and formed a more compact gel network, which consequently increased resistance to flow [64].

In contrast, the apparent viscosity of MHT-modified starch paste was significantly lower than that of native starch and decreased monotonically with increasing MHT duration. The decrease was attributed to the formation of thermally stable ordered structures, such as double helices, during MHT, as evidenced by the increased FTIR ratios and DSC T_p_. Although starch chains were also broken during MHT modification, the subsequent heat–moisture treatment facilitated the formation of these ordered structures. Due to their high thermal stability, these structures were not fully disrupted during gelatinization and thus did not contribute to gel network formation. Consequently, the resulting gel network was weaker, leading to lower apparent viscosity [64].

## 4. Discussion

Microwave-assisted heat–moisture treatment with low water content induced fragmentation and rearrangement of molecular chains in wheat starch. During the process, the microwave treatment heated water molecules via dipolar rotation, and the heat was subsequently transferred to starch chains, disrupting hydrogen bonds and facilitating chain degradation. The subsequent heat–moisture treatment further degraded starch chains through sustained thermal energy. The combination of the two treatments acted synergistically, providing conditions for starch chain rearrangement and ordered structure formation. Experimental results indicated that the local molecular order was enhanced after MHT, as reflected by the increased R_1050/1020_ and R_990/1020_ ratios. DSC results indicated a continuous increase in gelatinization peak temperature accompanied by a decrease in enthalpy for the modified starch, which suggested fewer long-range ordered structures but higher thermal stability in the samples. Furthermore, the increased double-helix content observed by FTIR likely formed a physical barrier that restricted enzyme access, thereby reducing starch digestibility, which is characteristic of RS3. Based on these structural changes, the resistant starch content reached a maximum of 36.89% after 1.5 h of MHT. Morphological analysis revealed that the modified starch granules exhibited progressively roughened and pitted surfaces, while the viscosity of the treated starch decreased continuously. In summary, the study demonstrated that targeted modification of starch digestibility through MHT can be achieved by precisely controlling its multi-level structural order, and thus provided a theoretical foundation for the production of highly resistant starch.

## Figures and Tables

**Table 1:** Short-ordered degree of native starch and modified starch samples.

Sample	R_1050/1020_	R_990/1020_
WS	1.45 ± 0.04 ^c^	1.21 ± 0.06 ^b^
MTS	1.58 ± 0.04 ^b^	1.55 ± 0.01 ^a^
MHT-0.5 h	1.59 ± 0.01 ^b^	1.25 ± 0.07 ^b^
MHT-1 h	1.63 ± 0.04 ^ab^	1.28 ± 0.02 ^b^
MHT-1.5 h	1.67 ± 0.05 ^a^	1.54 ± 0.10 ^a^
MHT-2 h	1.62 ± 0.02 ^b^	1.52 ± 0.03 ^a^

Values in the same column marked with different letters indicate significant differences at the *p* < 0.05 level.

**Table 2:** Thermal characteristic parameters of native starch and modified starch samples.

Sample	T_0_ (°C)	T_p_ (°C)	T_c_ (°C)	ΔT (°C)	ΔH (J/g)
WS	57.53 ± 0.31 ^d^	62.63 ± 0.32 ^f^	66.90 ± 0.9 ^c^	9.37 ± 0.73 ^ab^	9.52 ± 0.90 ^a^
MTS	57.17 ± 0.47 ^d^	61.93 ± 0.50 ^e^	66.77 ± 1.30 ^c^	9.60 ± 1.67 ^ab^	6.57 ± 1.29 ^bc^
MHT-0.5 h	57.30 ± 0.52 ^d^	64.80 ± 0.26 ^d^	69.03 ± 1.33 ^bc^	11.73 ± 1.85 ^a^	6.70 ± 0.15 ^b^
MHT-1 h	60.93 ± 0.64 ^c^	66.53 ± 0.35 ^c^	71.73 ± 1.97 ^ab^	10.80 ± 2.60 ^ab^	6.30 ± 0.41 ^bc^
MHT-1.5 h	63.87 ± 0.55 ^b^	69.10 ± 0.26 ^b^	71.83 ± 2.86 ^ab^	7.97 ± 2.30 ^ab^	5.43 ± 0.41 ^de^
MHT-2 h	67.13 ± 0.64 ^a^	70.97 ± 0.23 ^a^	74.50 ± 0.95 ^a^	7.37 ± 1.35 ^b^	4.82 ± 0.39 ^e^

Values in the same column marked with different letters indicate significant differences at the *p* < 0.05 level.

**Table 3:** In vitro digestibility of native starch and modified starch samples.

Sample	RDS (%)	SDS (%)	RS (%)	C_∞_ (%)	*k* (min ^−1^)	R^2^
WS	69.19 ± 0.58 a	18.89 ± 0.57 a	11.92 ± 1.00 e	92.64 ± 0.49 ^a^	0.0594 ± 0.0004 ^a^	0.95245
MTS	61.09 ± 0.58 b	13.50 ± 0.58 b	25.41 ± 1.17 d	80.21 ± 0.70 ^b^	0.0571 ± 0.0001 ^ab^	0.94646
MHT-0.5 h	55.35 ± 0.58 c	12.31 ± 1.27 bc	32.33 ± 1.78 c	73.72 ± 2.07 ^c^	0.0535 ± 0.0054 ^ab^	0.93512
MHT-1 h	55.01 ± 0.58 c	11.47 ± 1.17 c	33.50 ± 0.58 bc	72.47 ± 0.77 ^cd^	0.0547 ± 0.0026 ^ab^	0.93063
MHT-1.5 h	51.97 ± 0.58 e	11.14 ± 1.01 c	36.89 ± 1.17 a	69.78 ± 1.14 ^e^	0.0524 ± 0.0031 ^b^	0.92341
MHT-2 h	54.00 ± 0.59 d	11.47 ± 0.58 c	34.52 ± 1.17 b	71.81 ± 0.42 ^d^	0.0541 ± 0.0014 ^ab^	0.93192

Values in the same column marked with different letters indicate significant differences at the *p* < 0.05 level.

**Table 4:** Pasting properties of native starch and modified starch samples.

Sample	PV (cP)	TV (cP)	BD (cP)	FV (cP)	SB (cP)	PT (°C)
WS	1100.00 ± 7.94 ^a^	740.33 ± 4.72 ^a^	359.67 ± 5.03 ^a^	1252.33 ± 5.51 ^a^	512.00 ± 1.73 ^a^	82.30 ± 0.80 ^e^
MTS	656.67 ± 16.26 ^b^	381.67 ± 12.42 ^b^	275.00 ± 4.58 ^b^	668.00 ± 6.56 ^b^	268.33 ± 7.02 ^b^	84.72 ± 0.03 ^d^
MHT-0.5 h	370.33 ± 6.81 ^c^	204.33 ± 5.03 ^c^	166.00 ± 5.20 ^c^	424.67 ± 3.21 ^c^	220.33 ± 8.02 ^c^	86.13 ± 0.46 ^c^
MHT-1 h	345.00 ± 15.72 ^d^	194.00 ± 17.35 ^c^	151.00 ± 7.00 ^d^	399.33 ± 45.71 ^c^	205.33 ± 28.57 ^c^	87.20 ± 0.05 ^b^
MHT-1.5 h	297.67 ± 10.27 ^e^	164.00 ± 6.93 ^d^	133.67 ± 4.16 ^e^	319.00 ± 2.64 ^d^	155.00 ± 9.54 ^d^	88.27 ± 0.46 ^a^
MHT-2 h	206.00 ± 11.53 ^f^	109.00 ± 14.73 ^e^	97.00 ± 7.94 ^f^	217.33 ± 37.98 ^e^	108.33 ± 23.289 ^e^	87.43 ± 0.40 ^b^

Values in the same column marked with different letters indicate significant differences at the *p* < 0.05 level.

## Data Availability

The original contributions presented in this study are included in the article/Supplementary Material. Further inquiries can be directed to the corresponding authors.

## Supplementary Materials

The following supporting information can be downloaded at: https://www.mdpi.com/article/10.3390/foods15101698/s1, Figure S1: The DSC thermograms of native starch and treated starch samples. Figure S2: The RVA pasting curves of native starch and treated starch samples.
