# Supplementary material for: Effect of Microwave-Assisted Heat–Moisture Treatment on Structure, Physicochemical Properties and In Vitro Digestibility of Wheat Starch
Source: Foods. 2026 May 12;15(10):1698. doi: 10.3390/foods15101698 (PMC13205637; doi:10.3390/foods15101698)
Supplement: Supplementary file 1 [file foods-15-01698-s001.zip › foods-4292503-supplementary.pdf]

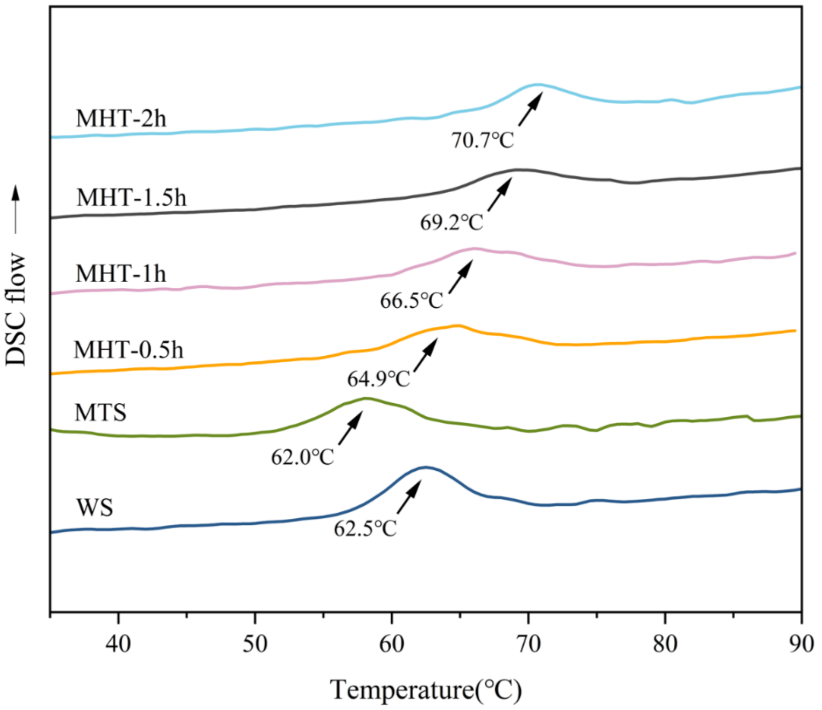

Figure S1 The DSC thermograms of native starch and treated starch samples.

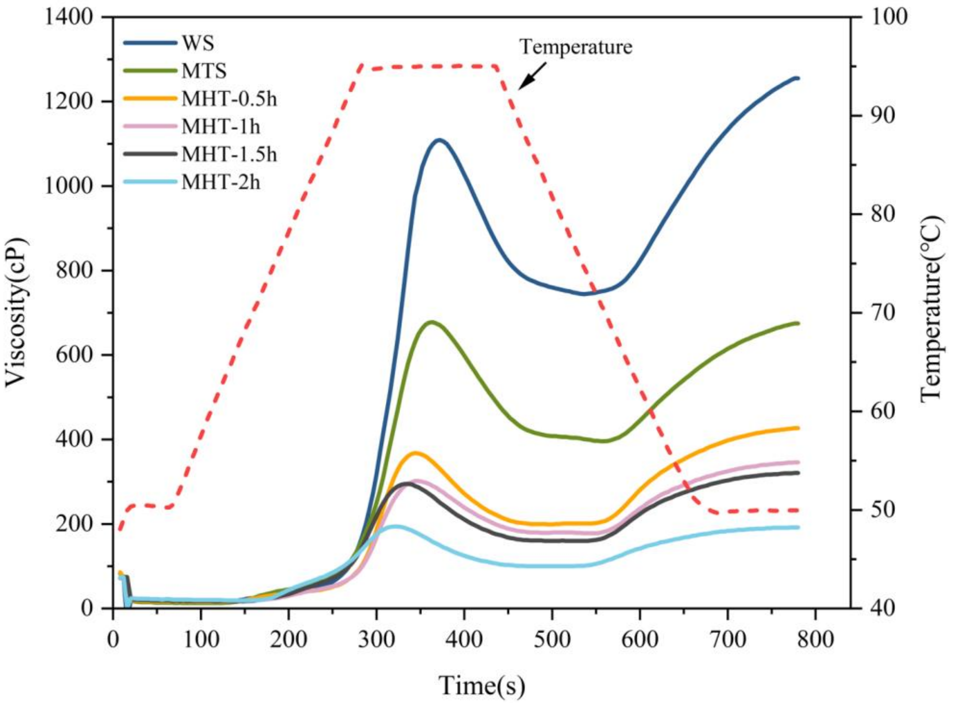

Figure S2 The RVA pasting curves of native starch and treated starch samples.
